# Transcriptome and Physiological Analysis Highlight Lignin Metabolism of the Fruit Dots Disordering during Postharvest Cold Storage in ‘Danxiahong’ Pear

**DOI:** 10.3390/genes14091785

**Published:** 2023-09-11

**Authors:** Ruiwei Duan, Xiangzhan Zhang, Yudong Liu, Lei Wang, Jian Yang, Long Wang, Suke Wang, Yanli Su, Huabai Xue

**Affiliations:** 1 Zhengzhou Fruit Research Institute, Chinese Academy of Agricultural Sciences, National Key Laboratory for Germplasm Innovation & Utilization of Horticultural Crop, Zhengzhou 450009, China; weiwei6182021@163.com (R.D.); zhangxiangzhan@caas.cn (X.Z.); ewlei@163.com (L.W.); yangjian@caas.cn (J.Y.); wanglong02@caas.cn (L.W.); wangsuke@caas.cn (S.W.); suyanli@caas.cn (Y.S.); 2 Key Laboratory of Fruit Breeding Technology of Ministry of Agriculture and Rural Affairs, Zhengzhou Fruit Research Institute, Chinese Academy of Agricultural Sciences, Zhengzhou 450009, China; 3 Henan Key Laboratory of Fruit and Cucurbit Biology, Zhengzhou Fruit Research Institute, Chinese Academy of Agricultural Sciences, Zhengzhou 450009, China; 4 School of Agricultural Sciences, Zhengzhou University, Zhengzhou 450001, China; yudongliu2022@163.com

**Keywords:** pear, fruit dots, protrusion, lignin metabolism, transcriptome

## Abstract

Pear (*Pyrus* L.) is one of the most important fruits in the world. Fruit dots are an important trait that affects pear quality. Abnormal fruit dots usually reduce the merchantability of pears. In this research, during cold storage, ‘Danxiahong’ pear fruit exhibited protrudent fruit dots on the peels. Microscopy system measurement showed that fruit dots size and height on the abnormal fruit peel were bigger and higher than the normal ones. Likewise, scanning electron microscopy observations indicated that the abnormal fruit peel, in contrast to the normal pear peel, exhibited an abnormal cell structure and fruit dots. Physiological analysis showed that the lignin content in abnormal fruit peel was significantly higher than in normal fruit peel. Gene Ontology and Kyoto Encyclopedia of Genes and Genomes enrichment analysis revealed that the top-enriched pathways were mainly associated with lignin synthesis and metabolism. The transcripts of lignin biosynthesis-associated genes were analyzed, and the results showed that the expression of a cascade of structural genes, including *PpyPAL*, *PpyCCR*, *PpyC3H, PpyC4H, PpyHCT*, *PpyCAD*, *PpyLAC*, and *PpyPOD*, was significantly induced in the protrudent peels. Furthermore, the expression of regulatory genes involved in lignin biosynthesis, especially the NAC-MYB-based gene regulatory network, was significantly upregulated in the abnormal peels. Real-time quantitative PCR (RT-qPCR) analysis confirmed the induction of lignin biosynthesis genes. Overall, this research revealed that the abnormal fruit surface was caused by fruit dots disorder during cold storage. This research provides insights into the fine regulation pathways in the prevention of fruit dots protrusion, especially in modulating lignin synthesis and metabolism during postharvest storage.

## 1. Introduction

Pear (*Pyrus* L.), one of the most important fruits, is widely cultivated worldwide and is an economically important fruit in China [1]. Fruit dots are an important characteristic of pear peels, affecting pear fruit quality, especially for pears originating from East Asia [2,3]. A noticeable protrusion of fruit dots usually occurs in fruit peels of some pear cultivars during long-terms cold storage, which leads to massive economic losses [4]. It is of great significance to reveal the physiological basis and molecular mechanism underlying abnormal fruit dot disorder on the fruit peel.

Fruit dots are mainly formed with the development of stoma on fruit peels [5]. The stoma on pear peels breaks during fruit development, and the parenchyma in the stoma is divided into the phellogen. Finally, the phellogen produces cork cells that gradually appear on the fruit skin, and the fruit lenticels form, indicating that fruit dot formation is related to cork cell production and lignification formation [6]. Studies also indicated that lignin and other phenolic substances are structural components of cork cells [7,8]. However, the physiological changes underlying fruit dots formation during cold storage are largely uncovered.

During pear fruit development, the formation of fruit dots is affected by genetic factors and various external factors, including pesticides, dust, smoke, pathogenic microorganisms, environmental stimuli, and other stress factors [9]. Preharvest bagging contributed to the improvement of fruit dots, which resulted in smooth fruit peels with smaller fruit dots and less protrusion [10,11,12]. However, few reports have focused on the molecular mechanism and transcriptional changes underlying disordered fruit dots during long-term cold storage.

In plants, lignin is a principal structural component of cell walls that plays an important role in various biological processes, including water retention, mechanical support, and protection [13]. Moreover, the lignification process in fruit plays an important role in the abiotic stress response and defense against pests and diseases [3,14]. The lignin biosynthesis pathway has been well elucidated in plants, and many structural genes encode enzymes that are involved in lignin biosynthesis [15]. Growing evidence indicated that lignin accumulation is consistent with the transcript expression of structural genes, such as phenylalanine ammonia-lyase (PAL) [16], Cinnamoyl CoA reductase (CCR) and cinnamyl alcohol dehydrogenase (CAD)genes [17,18]. Studies have shown that *PbCAD* and *PbCCR* genes participate in lignin synthesis in the stone cells in pear fruit [19]. 

Apart from structural genes, regulatory genes have also been reported to mediate lignin biosynthesis, among which NAC and MYB transcription factors have been extensively reported [20,21]. The NAC-MYB-based gene regulatory network (NAC-MYB-GRN) model is considered to be involved in lignin biosynthesis [22]. The crucial role of MYB transcription in lignin accumulation has been well documented in diverse plant species, including *Arabidopsis* (*Arabidopsis thaliana* L.), grape (*Vitis vinifera* L.), loquat (*Eriobotrya japonica* L.), and pear (*Pyrus* L.) [23,24,25,26,27]. Several studies have demonstrated that NAC transcription factors serve as master switches in the lignin biosynthesis pathway [28,29,30,31]. In ‘Whangkeumbae’ pear, *PpNAC187* functions to increase lignin accumulation by inducing the expression of *PpCCR* and *PpCOMT* during fruit development [30]. In addition, other types of regulatory factors, such as WRKY transcription factors [32,33], bHLH transcription factors [34], and BZR transcription factors [35], have also been reported to be involved in lignin biosynthesis. In Chinese pear (*Pyrus bretschneideri*), *PbBZR1* represses the transcription of lignin biosynthesis genes, including *PbCES9*, *PbCOMT3*, and *PbHCT6* [35], indicating the role of BZR transcription factor mediating the biosynthesis of lignin.

Until now, few reports have focused on the relationship between protrusions of pear fruit dots and lignin synthesis during cold storage. In this research, during long-term cold storage, pear cultivar ‘Danxiahong’ exhibited an abnormal appearance with enlarged and protruding fruit dots on fruit peels. Microscopic observation and physiological analysis indicated that the protrusions of pear fruit dots were caused by the lignin biosynthesis Further transcriptome analysis show a large number of differential genes enriched in the lignin metabolic pathway, many structural genes were upregulated in the lignin biosynthesis pathway. Six MYB transcription factors, three NAC transcription factors and seven WRKY transcription factors were upregulated. RT-qPCR analysis verified the induction of lignin biosynthesis genes. This study revealed the lignin metabolism underlying fruit dots disordering during postharvest cold storage in ‘Danxiahong’ pear by transcriptome and physiological analysis.

## 2. Results

### 2.1. ‘Danxiahong’ Pear Fruit Dots Morphology and Lignin Content

**Figure 1:**
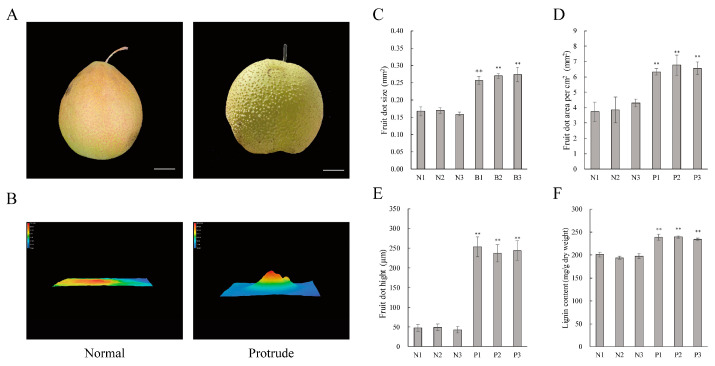
Phenotypic observation and measurement of fruit dots on ‘Danxiahong’ pear. ‘Danxiahong’ pear fruit with a normal (**A**) and protrudent (**B**) appearance during cold storage at 0 °C for three months, scale bar indicates 5 cm. Determination of fruit dot size (**C**), area (**D**), and height (**E**) of normal and protrudent pear peels. Measurement of lignin content in ‘Danxiahong’ pear (**F**). Asterisks indicate statistical significance (**, *p* < 0.01) calculated by Student’s *t*-test.

The peel of ‘Danxiahong’ pear fruits displayed evident protrusions and exhibited an abnormal appearance after storage at 0 °C for three months (Figure 1A), which significantly reduced the commercial value. To investigate the possible morphological alteration for the protruding fruit dots on ‘Danxiahong’, the pear peels with normal and protruding fruit dots were sampled and observed under a KEYENCE VHX-6000 Digital microscope. Microscopic observation showed that the abnormal peels exhibited significant protrudent fruit dots (Figure 1B). In the abnormal peels, there was a clear circle of wax around the fruit dots, and the height of the fruit dots showed a noticeable increase (Figure S1). A Keyence microscope was used to simulate the fruit dots in the 3D model, and the fruit dots protruded significantly (Figure 1B). 

To further investigate the morphological change, the fruit dot size, fruit dot area per unit area, density, and fruit dot height were determined. The protrudent fruit peel exhibited a larger fruit dot size than the normal peels (Figure 1C). The fruit dot area per unit area and fruit dot height on protrudent fruit peels were significantly higher than on normal peels (Figure 1D,E). However, the fruit dot density showed no significant difference between normal and protrudent fruit (Figure S1). The lignin content was investigated, and the results revealed that pears that exhibited protrudent fruit dots had a higher lignin content than the normal fruit (Figure 1F). The results indicate that during cold storage, ‘Danxiahong’ pear exhibits a rough peel surface, which may associate with lignin biosynthesis.

### 2.2. Scanning Electron Microscopy and Paraffin Section Observation 

**Figure 2:**
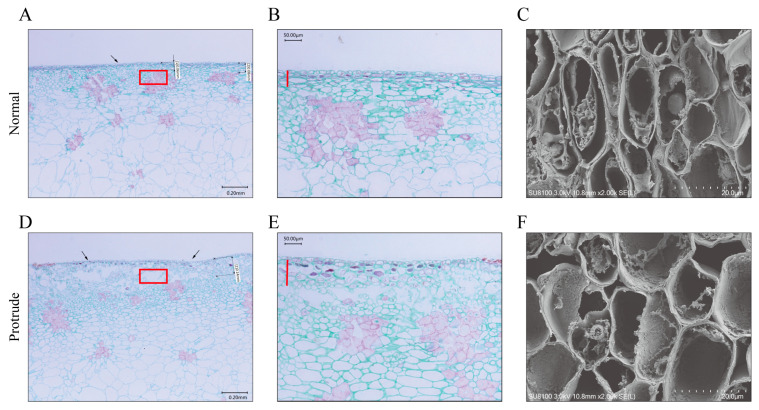
Micromorphology observation of normal fruit dots (**A**–**C**) and protruded fruit dots (**D**–**F**) in ‘Danxiahong’ pear. (A/D: 30× magnification, scale bar indicates 20 μm; B/E: 50× magnification, scale bar in Figure 2A,D indicates 50 μm under the field of 50× magnification) and scanning electron microscopy (2000× magnification, scale bar indicates 20 μm). The red circle and bar describe the differences in cork cells between normal and protrudent samples. The arrows indicate the differences in cuticle between normal and protrudent samples.

Given the morphological alteration in fruit dots between normal and abnormal pear peels, we next investigated the tissue structure underlying pear peels. Paraffin sections showed that the surface layer of the abnormal peel was bulging; however, the outer cuticle of the normal peel was intact, and the lower epidermis was tightly constructed without holes (Figure 2A–E). The epidermal cell gap of the abnormal fruit was significantly larger than that of the normal fruit (Figure 2A–E). In addition, some of the epidermal cells in the protrudent peel were deep purple, and the number of deep purple cells was higher than in normal fruit epidermal cells (Figure 2B,E). This indicates that the lignin content of pro-trudent peels increased. Scanning electron micrographs showed the closeness of the epidermal cells; the epidermal cells were closer together in normal peels than in abnormal peels (Figure 2C,F).

### 2.3. RNA Sequencing and Analysis of Differentially Expressed Genes

**Figure 3:**
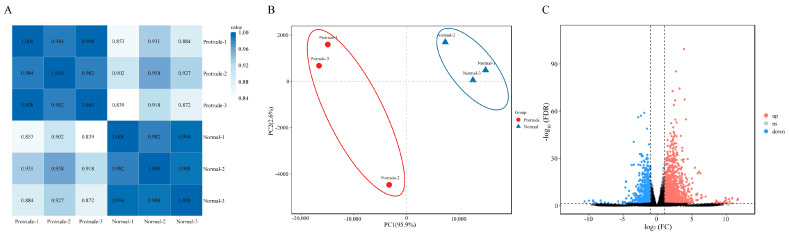
Correlation analysis and DEG analysis of normal and protrudent peel samples. (**A**) Pea-son’s correlation coefficient analysis of sample with normal and protruded peels. The blue scale indicates high correlation, and white scale indicates low correlation. (**B**) Principal component analysis of the samples with normal and protruded peels. The blue circle indicates clustered into one category, and the red circle indicates clustered into one category. (**C**) Volcano plot of DGEs between normal and protruded samples. Red dots indicate up regulated genes, blue dots indicate down regulated genes, and gray dots indicate nonsignificant differential expressed genes.

To investigate the transcript changes underlying fruit dot protrusion, normal and protrudent ‘Danxiahong’ pear peels were sampled and subjected to RNA-seq analysis. A total of six cDNA libraries, representing three replicates from each group (normal-1, normal-2, normal-3, protrude-1, protude-2, and protude-3), were constructed for high-throughput sequencing. Overall, 20.4 GB of clean data were obtained, and an average of 46,857,469 clean reads were obtained, with a Q20 quality score ≥ 93.68% and a Q30 quality score ≥ 93.88%, indicating the high quality of the data (Table 1). The clean reads were aligned with the pear genome (*Pyrus pyrifolia* (Burm.f.) Nakai) [35], and the average mapping percentage reached 92.81% (Table 1). The correlation between different samples was analyzed, and all samples were grouped into two distinct types. Samples belonging to the same type exhibited a high correlation (Figure 3A). PCA showed clear separation between the two groups, and the samples in the same group showed a close association (Figure 3B). Genes with |log2FC| > 1 and FDR < 0.05 were considered significant DEGs, and a total of 1960 DEGs were identified between the normal and protrudent groups, including 1252 up- and 708 downregulated genes (Figure 3C). These results indicated the reliability of the experimental samples and data, which were then used for further analyses.

### 2.4. Gene Ontology (GO) Enrichment Analysis of DEGs

**Figure 4:**
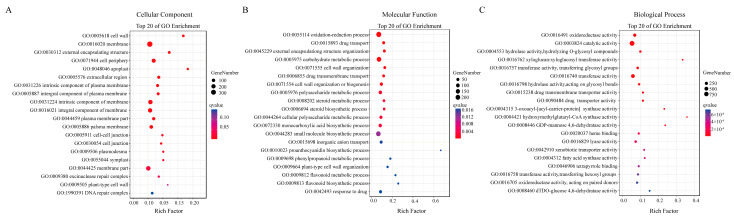
Gene Ontology (GO) enrichment analysis of DEGs between normal and protrudent samples. Y-axis indicates top-twenty enriched items, X-axis indicates the enrichment factor. The circle size indicates the number of DEGs, and the color scale indicates the q value, in which the red color indicates low q value. (**A**–**C)** indicate three ontology of GO that describe molecular function cellular component and biological process, respectively.

To obtain further information on the potential roles of the DEGs identified between the normal and protrudent samples, GO enrichment analysis was conducted based on the RNA-seq data. Result revealed that the DEGs were mainly classified into three categories: “Cellular Component”, “Molecular Function”, and “Biological Process” (Figure 4A–C). In the category of “Cellular Component”, a cascade of DEGs was significantly enriched in different items, including “cell wall”, “membrane”, “external encapsulating structure”, and “cell junction” (Figure 4A), indicating the roles of enriched DEGs function in the cell wall, plasma membrane, cell junction, and other subcellular structures. In the category of “Molecular Function”, the DEGs were significantly enriched in the items of “oxidation-reduction process”, “external encapsulating structure organization”, “carbohydrate metabolic process”, “cell wall organization”, “polysaccharide metabolic process”, and “phenylpropanoid metabolic process” (Figure 4B). These molecular function processes were mainly associated with cell wall structure organization and the phenylpropanoid metabolic process, which may have caused the protrudent pear peels. 

In the “Biological Process” category, GO terms of “oxidoreductase activity”, “catalytic activity”, “xyloglucosyl transferase activity”, “lyase activity”, and “fatty acid synthase activity” were significantly enriched (Figure 4C). The DEGs enriched in these items may have been related to the change in lignin content in ‘Danxiahong’ pear peel.

Additionally, many GO terms reflected a response to cell wall, membrane, and secondary metabolic processes, including “xyloglucan: xyloglucosyl transferase activity”, “anchored component of membrane”, “phenylpropanoid metabolic process”, “flavonoid biosynthetic process”, and “dTDP-glucose 4,6-dehydratase activity”, (Figure 4A–C) which confirmed the transcript expression of genes involved in lignin biosynthesis and metabolism in pear peel.

### 2.5. KEGG Enrichment Analysis of DEGs

**Figure 5:**
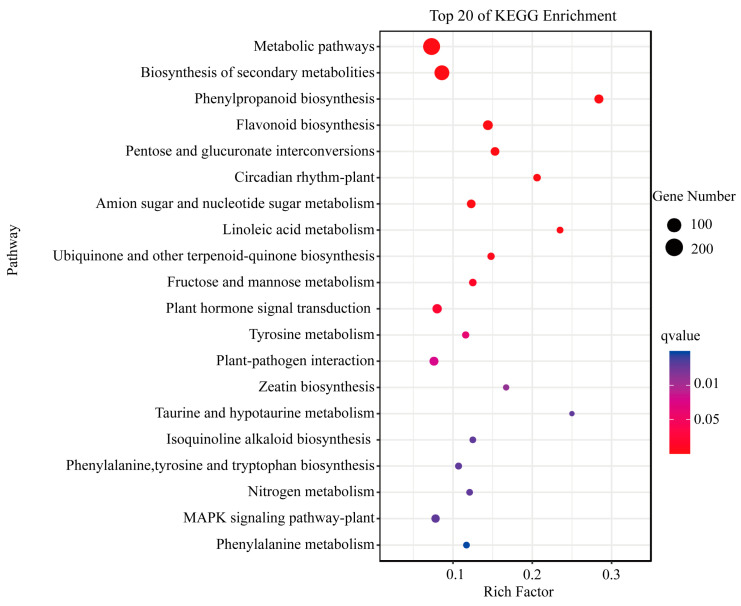
KEGG enrichment analysis of DEGs. Y-axis indicates top-20 enriched items, X-axis indicates the enrichment factor. The circle size indicates the number of DEGs, and the color scale indicates the q value, in which the red color indicates high q value.

To further investigate the possible molecular functions of the DEGs in the normal and protrudent pear peels of ‘Danxiahong’, KEGG enrichment analysis was performed (Figure 5). Result showed that the DEGs were significantly enriched in a number of pathways, including “metabolic pathways”, “biosynthesis of secondary metabolites”, “phenylpropanoid biosynthesis”, “flavonoid biosynthesis”, “tyrosine metabolism”, and “phenylalanine metabolism” (Figure 5), indicating the possible involvement of these DEGs in the pathways associated with fruit dot disorder in pear peels. Additionally, many KEGG terms including “Tryosine metabolism”, “Phenylalanine, tyrosine and tryptophan biosynthesis” and other items, were also significantly enriched, implying the possible involvement of the DEGs in these pathways that resulted in the protrudent pear peels. 

### 2.6. Analysis of Lignin Biosynthesis Structural Genes

**Figure 6:**
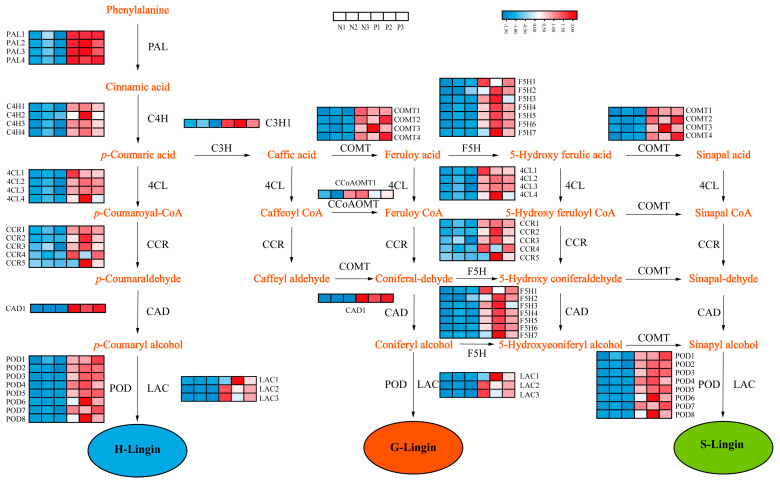
Heatmap of the expression profiles of structural genes involved in lignin biosynthesis. The color scale indicates the gene expression levels. Red indicates high expression and blue indicates low expression.

Considering the differences in lignin content between the normal and protrudent pear peels and the enriched pathways that were mainly associated with cell wall organization and phenylpropanoid biosynthesis, the lignin biosynthesis genes were analyzed (Table S1). Lignin biosynthesis is regulated by multiple structural genes, and the well-annotated phenylpropanoid biosynthesis pathway involved in lignin biosynthesis was analyzed using RNA-seq data. Structural genes encoding key enzymes involved in the lignin biosynthesis pathway, including PAL, C4H, 4CL, CCR, C3H, COMT, CAD, PPO, and LAC, were identified (Figure 6). The transcript abundance of some structural genes at the earlier stage of lignin biosynthesis, including members of PpyPALs, PpyC4Hs, Ppy4CLs, and PpyCCRs, exhibited significantly induced transcript levels in the protrudent peels (Figure 6). The structural genes at the middle and later stages of lignin biosynthesis, including most members of PpyF5Hs, PpyLACs, PpyCOMTs, and PpyPODs, exhibited similar and significantly upregulated transcript levels (Figure 6). Most members in the phenylpropanoid biosynthesis pathway exhibited induced transcript level in the abnormal pear peels, although a few members showed different transcript patterns (Figure 6), indicating the diverse roles of the structural genes. Altogether, these results indicated that protrdent fruit dots are associated with altered gene related with lignin biosynthesis expression.

### 2.7. Analysis of Lignin Biosynthesis Regulatory Genes

**Figure 7:**
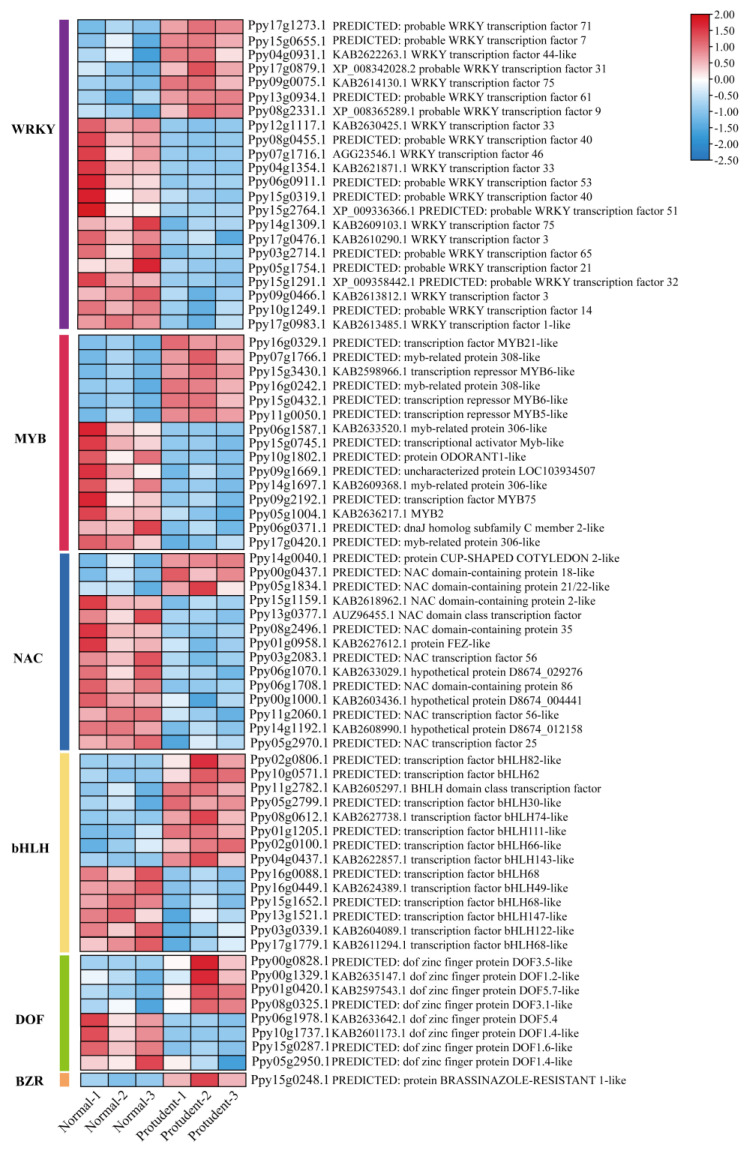
Expression profiles of different transcription factor families between the normal and prot-rudent pear peels. Heatmap was performed using the TBtools with the data normalized to log scale and row scale. Red indicates high expression level and blue indicates low expression level.

Increasing evidence indicates that lignin biosynthesis is regulated by different transcription factors in diverse plant species [33,34,35]. To investigate the potential transcription factors involved in lignin biosynthesis, the transcript levels of a set of transcription factors between normal and protrudent pear peels were investigated (Table S2). The WRKY family was analyzed, and seven WRKY genes exhibited significant upregulation in protrudent pear peels (Figure 7). Moreover, many MYB transcription factors, including MYB5, MYB20, and other MYBs, were significantly induced in protrudent pear peels, indicating the possible roles of these MYB genes involved in fruit dot regulation in protrudent pear peels. NAC family transcription factors were analyzed, and some NAC members, including NAC047, NAC021, and NAC087, exhibited significant upregulation in protrudent pear peels. In addition, bHLHs annotated with bHLH82, bHLH62, bHLH30, bHLH74, bHLH111, and bHLH66, as well as DOF transcription factors annotated with DOF3.5, DOF3.1, DOF1.2, and DOF5.7, were significantly induced in protrudent pear peels. One BZR transcription factor showed significant induction in protrudent pear peels, indicating the possible involvement of these transcription factors in fruit dot disorder-meditated lignin accumulation. Additionally, some transcription factors exhibited repressed transcript level in the protrudent pear peels, including some WRKY, MYB, NAC, bHLH and DOF transcription factors (Figure 7), indicating the different role of these transcription factors mediating lignin biosynthesis during cold storage.

### 2.8. RT-qPCR Analysis of the Lignin Biosynthesis Genes

**Figure 8:**
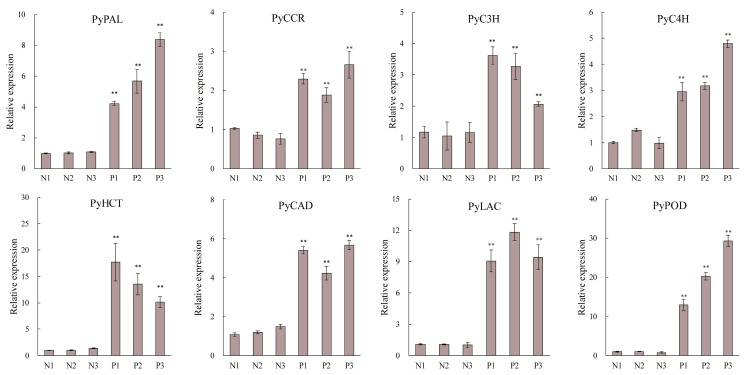
RT-qPCR analysis of lignin biosynthesis structural genes. N1, N2, and N3 indicate three biological replicates from normal phenotype pear; P1, P2 and P3, indicate three biological replicates from protrudent pear peels. Error bars indicate the mean ± SD of three independent replicates. Asterisks indicate statistical significance (**, *p* < 0.01) calculated by Student’s *t*-test.

To verify the expression of the lignin biosynthesis genes obtained from the RNA-seq data, several structural genes involved in lignin biosynthesis, including PAL, CCR, C3H, C4H, HCT, CAD, LAC, and POD, were selected for RT-qPCR analysis. Result showed that the transcript levels of these genes were significantly induced in the protrudent samples (Figure 8). Among them, the expression levels of PyHCT and PyPOD in protrudent peels were induced 10 times more than those in normal peels. Additionally, some lignin biosynthesis structural genes, including PyLAC, PyCAD, PyC3H, PyPAL, and PyCCR, were significantly induced in the protrudent samples. Taken together, these results suggest that fruit dot disorder in ‘Danxiahong’ was mainly associated with transcript upregulation of the genes related to lignin biosynthesis.

## 3. Discussion

A physiological disorder on the pear fruit peel of ‘Danxiahong’, here named fruit dot disorder, usually occurred after long-term cold storage, which resulted in an abnormal peel appearance with enlarged and protrudent fruit dots (Figure 1). Further determination and observation with digital microscope showed that the fruit dots from abnormal pear had larger and higher value, and the lignin content significantly higher than normal fruit peel. This was similar to the study of ‘Xinli No. 7′ [4]. The normal fruit dots exhibited a typical “moon-crater”-type appearance with well-defined edges and a smooth peel, which was consistent with study of ‘Clapp’s Favourite’ and ‘Conference’ [36]. Here, our observation with scanning electron microscopy (SEM) and paraffin section showed that the surface layer of the abnormal peel was bulging, and the lower epidermis was disrupted with holes (Figure 2), which was rarely reported previously, and no regulatory mechanisms have been characterized associated with the phenomenon. Staining of lignin in normal or abnormal fruit peel structures using safranin O-fast green, with abnormal fruit staining more deeply staining. This result was in agreement with the results of lignin content determination (Figure 1). were caused by the lignin biosynthesis. These results implied that the abnormal pear peels are likely to be caused by inappropriate lignin biosynthesis.

Microscopic observation showed that the protrudent fruit dots bulged and a white ring around the fruit dot (Figure S1), which was possible the wax on the peel surface, while the normal peels did not protrudent. The surface wax on fruit has a significant effect on abiotic stress and fruit quality [37]. The cuticular wax in fruit contributes to the restriction of water loss, which also affects stoma development in response to various environmental conditions [38]. Previous studies have shown that epidermal fruit wax undergoes significant changes during storage, and that a good wax layer protects the integrity of fruit during storage [39]. Thus, it was suspected that epidermal wax may be associated with the protective mechanism during cold storage. Additionally, KEGG analysis showed that some DEGs were enriched in “Linoleic acid metabolism”, “Phenylalanine, tyrosine and tryptophan biosynthesis”, and “Pentose and glucuronate interconversions”, (Figure 4) which are associated with the biosynthesis of epidermal wax. This implies that the possible connection of fruit dot disordering and wax biosynthesis in pear peels in response to abiotic stress during storage.

It was showed that the color of abnormal pear peel changed to yellow, while the normal pear peel showed slight yellow during cold storage (Figure 1). indicating the different ripening conditions of the pear fruits, this was similar to the study of ‘Xinli No. 7′ that the mature condition affect the peel appearance [4]. Therefore, the result implies that the storage conditions, different maturities, as well as the inner and outer chambers of the trees may affect lignin synthesis in response to cold storage. Additionally, we found that most European pear cultivars and some Asian pears did not exhibit bulging fruit dots after long-term cold storage, indicating the inner genetic factor regulating this characteristic. Apart from this, other micro-enviroments, including CO_2_, H_2_O, and ethylene, may also have an impact on the protrudent fruit dots during cold storage, and more investigation is needed to support this speculation.

Low temperatures usually promote lignification in plants, such as in loquat and kiwifruit, the lignin accumulation usually leaded to the lignification, which is the main reason for the morphological changes in fruit appearance during cold storage [40,41]. Our study indicates that the abnormal fruit exhibited a significantly higher lignin content than the normal fruit (Figure 1F), which is consistent with previous reports in pear and other plant species [4,40,41]. Combined with the induction of most lignin biosynthesis structural genes and regulatory genes in abnormal pear peels, as well as the enriched GO and KEGG items associated with lignification; therefore, it is possible that the cold storage was associated with the biosynthesis of lignin. Apart from the phenylalanine metabolic pathway, lignin biosynthesis is generated from “fatty acid synthase activity” and “tyrosine metabolism”, which are complex secondary metabolic processes. These results indicate that long-term cold storage promoted fruit lignification in pear peels, which was similar to previous study [42].

Lignin biosynthesis pathway has been well elucidated in plants; many structural genes encode enzymes that are involved in lignin biosynthesis [15]. PAL as a critical enzyme regulating lignin accumulation [43], and growing evidence indicated that lignin accumulation is consistent with the transcript expression of PAL genes [44,45]. Our result showed that all PAL genes exhibited significantly induced and similar transcript patterns in the abnormal and normal pear peels (Figure 6), indicating the role of PALs in the modulation of lignin biosynthesis. Moreover, CCR and CAD are involved in the monolignol synthesis, and overexpression of *PpCAD2* in tomato increases lignin deposition [46,47,48]. In addition, lignin synthesis also was regulated by 4-coumarane-CoA ligase (4CL), coumarate 3-hydroxylase (C3H), peroxidase (POD) and laccase (LAC), for lignin polymerization [49,50,51]. In our study, transcript analysis of the lignin biosynthesis structural genes showed induced expression in abnormal fruit peels (Figure 6). Meanwhile, structural genes encoding key enzymes, such as *PpyPALs*, *PpyC4Hs*, *Ppy4CLs*, and *PpyCCRs*, involved in lignin biosynthesis pathways were verified by RT-PCR analysis (Figure 8). The results further revealed that these genes were significantly induced in protrudent peels, verifying that the abnormal pear peels were caused by a disorder in lignin biosynthesis. In addition, six MYB transcription factors, three NAC transcription factors and seven WRKY transcription factors were upregulated (Figure 6). Thus, it was speculated that the transcription factors controlling lignin biosynthesis by regulating structural genes, which finally caused fruit dot disorder in pear peels.

## 4. Materials and Methods

### 4.1. Plant Materials and Experimental Treatments

The pear cultivar ‘Danxiahong’ planted in the orchard of Zhengzhou Fruit Research Institute, Chinese Academy of Agricultural Sciences, was used as the experimental material. Pear fruit was sampled in August 2022 at the mature stage and transferred to storage at 0 °C for preservation. Pears with normal and protruding peels were sampled after storage for about three months, and the materials were subjected to determine fruit dot size, density, height, and area measurement. Three biological replicates including eighteen pears were tested for the physiological measurement. Some of the indicated peels were sampled and immediately treated with liquid nitrogen for further lignin content determination, RNA-sequencing (RNA-seq) analysis, and real-time quantitative PCR (RT-qPCR) analysis.

### 4.2. Morphological Index Determination

Pear peels at the equator zone and on the sunny side were sampled with a sterile blade. The peel sample was placed on a slide and viewed under a KEYENCE-VHX-6000 digital microscope in a 100-fold visual field (KEYENCE-VHX-6000, Shanghai, China) to determine fruit dot size, density, and area. The fruit dots were photographed with a microscope, and the fruit dot size was determined using plane measurement in VHXMENU. Forty fruit dots were measured for each pear. The fruit dot density was calculated by counting the number of fruit dots per unit area, as described previously [2]. Twenty fields of view were measured for each pear. The fruit dot area was represented by the sum of the fruit dot areas within the fruit surface unit. The 3D model of fruit dots was synthesized with the 3D synthesis option in the digital microscope system with a 500-fold view using a KEYENCE-VHX-6000 digital microscope, and the fruit dot height was determined. The fruit surface was set as a horizontal line; if the fruit dot protruded from the fruit surface, then it was marked as positive, and if the fruit dot sank into the fruit surface, it was marked as negative. Ten fruit points were measured for each pear fruit. Three biological replicates were performed, and each index was determined for nine fruits.

### 4.3. Preparation and Observation of Paraffin Sections

Paraffin sectioning was performed following a previously described method [52]. Pear peels with a thickness of no more than 0.2 cm were sliced from normal and protruding pear fruits. The samples were cut into 0.3 cm × 0.3 cm pieces, fixed with FAA fixing solution, and then incubated in 70% ethanol solution for 12 h. The peel samples were dehydrated consecutively in 80% ethanol for 60 min, 90% ethanol for 30 min, and 100% ethanol for 15 min. The dehydrated samples were further incubated sequentially in anhydrous ethanol/xylene solution for 15 min, in xylene solution for 3 min, and in paraffin/xylene for 30 min, followed by paraffin embedding for 80 min. The embedded samples were sliced into sections with a thickness of 5 μm. The paraffin slices were transferred to slides with a toothpick, developed with warm water at 35 °C, placed on a slide coated with a thin layer of protein glycerin, and dried in an oven at 38 °C. The samples were treated with safranin O-fast green staining and sealed with neutral glue.

### 4.4. Scanning Electron-Microscopy (SEM) Observation

Fruit tissues with normal and protruding peels were sampled and immediately fixed in an electron microscopy fixative for 2 h at room temperature. The tissue blocks were washed with 0.1 M PB three times for 15 min each time. Then, the tissues were blocked with 30, 50, 70, 80, 90, and 95% ethanol for 15 min, with two changes of 100% ethanol for 15 min. Finally, isoamyl acetate was used for 15 min. Then, the samples were dried with a Critical Point Dryer (K850, Quorum, San Jose, CA, USA). Specimens were attached to metallic stubs using carbon stickers and sputter-coated (MC1000, HITACHI, Tokyo, Japan) with gold for 30 s. The images were captured using a scanning electron microscope (SU8100, HITACHI).

### 4.5. Lignin Content Determination

The lignin content was determined as described previously using a lignin extraction kit from Suzhou Michy Biomedical Technology Co., Ltd. (Suzhou, China). A total of eighteen pears including three biological replicates were tested. Briefly, the samples were pasteurized at 105 °C for 15 min, oven-dried to a constant weight at 80 °C, crushed, sieved, and weighed to obtain approximately 0.01 g of powdered sample. Then, 1 mL of 80% ethanol was added to a 2-mL EP tube and vortexed. The mixture was incubated in a water bath at 50 °C for 20 min and then centrifuged at 12,000 rpm for 10 min at 25. The supernatant was discarded, and the precipitate was retained and dried for later use. The absorbance was determined at 280 nm with a SpectraMax i3x Multi-Mode Detection Platform (Molecular Devices, San Jose, CA, USA). Iced acetic acid was set as the corresponding blank. Three biological replicates were performed, and each replicate included six samples. The lignin content was determined as follows: lignin content (mg/g dry weight) = (ΔA − 0.0388)/0.7337/W = 1.363 × (ΔA − 0.0388)/W, where W is the sample weight (g) and ΔA is A_determination_ − A_blank_.

### 4.6. Transcriptome Analysis with RNA-Seq Data Validation

Total RNA was isolated from pear peel with a Fast Plant RNA Kit for Polysaccharides & Polyphenolics-Rich according to the manufacturer’s instructions (Zomanbio, Beijing, China). The quality of the total RNA was examined using agarose gel electrophoresis and assessed using an Agilent 2100 Bioanalyzer (Agilent Technologies, Palo Alto, CA, USA). The cDNA libraries were sequenced on the Illumina sequencing platform by Genedenovo Biotechnology Co., Ltd. (Guangzhou, China). Raw reads were further filtered following Chen et al. [53]. The clean reads were mapped to the *Pyrus pyrifolia* Whole Genome v1.0 (https://www.rosaceae.org/, accessed on 11 January 2021) using HISAT2.2.4 [54].

### 4.7. DEG, GO, and KEGG Analysis

The expression abundance and the variation of each gene were calculated using the fragment per kilobase of transcript per million mapped reads (FPKM) method in RSEM software [55]. The difference in transcript abundance of the indicated gene between the two groups was analyzed using DESeq2 software [56]. Genes with |log2 (fold change)| > 1 and false discovery rate (FDR) < 0.05 were identified as differentially expressed genes (DEGs). The DEGs between the groups were visualized in a volcano plot using R software. Gene ontology (GO) enrichment analysis of DEGs was performed based on the GO database (http://www.geneontology.org/, accessed on 11 December 2022). Kyoto Encyclopedia of Genes and Genomes (KEGG) pathway enrichment analysis was conducted with the DEGs based on the KEGG database (https://www.kegg.jp/, accessed on 11 December 2022). A *p* value < 0.05 was considered as the cut-off criterion.

### 4.8. RNA Isolation and RT-qPCR Analysis

Total RNA was isolated using the Fast Plant RNA Kit for Polysaccharides& Polyphenolics-Rich (ZOMANBIO, Beijing, China) as described previously [57]. First-strand cDNA was synthesized using TransScript One-Step gDNA Removal and cDNA Synthesis SuperMix (TransGen Biotech, Beijing, China). RT-qPCR was performed using PerfectStart Green qPCR SuperMix with the Roche LightCycler 480 system (Roche, Basel, Switzerland) following the manufacturer’s instructions. The total reaction volume was 20 μL, and the amplification procedure was as follows: initial denaturation at 95 °C for 30 s and 40 cycles of denaturation at 95 °C for 5 s, annealing at 58 °C for 15 s, and extension at 72 °C for 34 s. The PcTubulin gene was used as the internal control, and the relative transcript level was analyzed using the 2^−∆∆Ct^ method [58]. Expression analysis of each gene in this research was repeated three times. The specific primers used in this research are listed in Table S3.

### 4.9. Principal Component Analysis

In this study, principal component analysis (PCA) was performed using the R package model to determine the correlation between samples [57].

### 4.10. Data Analysis

Statistical analysis was performed using Microsoft Excel 2010. Graphs were obtained from GraphPad Prism software and TBtools [59]. The values in each figure are the mean ± SD of three replicates. Significant differences were determined using Student’s *t*-test, and differences at *p* < 0.05 (*) and *p* < 0.01 (**) were labeled using the statistical tests.

## 5. Conclusions

Postharvest cold storage with long terms resulted in fruit dot disorder in ‘Danxiahong’ pear peel. The abnormal pear peels showed enlarged and bulging fruit dots and increased lignin content. The pathways including “phenylpropanoid biosynthesis” and other cell wall organization-associated items were significantly enriched. The expression of structural genes and transcription factors associated with lignin biosynthesis was induced in the protrudent peels. RT-qPCR analysis verified the transcript induction of lignin biosynthesis structural genes. Collectively, this study revealed a physiological disease in pear peels that was mainly caused by inappropriate lignin biosynthesis and metabolism. This research also provides a theoretical basis for elucidating the relationship between fruit dot disorder and lignin biosynthesis in ‘Danxiahong’ pear.

## Figures and Tables

**Table 1:** Statistics of sequencing data of all libraries.

Sample	Raw Data	Clean Data (%)	Q20 (%)	Q30 (%)	GC (%)	Total Mapped (%)
Normal 1	44,838,532	44,699,178 (99.69%)	97.86%	93.75%	46.45%	41,436,589 (92.77%)
Normal 2	53,906,118	53,762,512 (99.73%)	94.04%	94.04%	46.51%	49,783,567 (92.68%)
Normal 3	47,423,092	47,287,074 (99.71%)	97.92%	93.86%	46.56%	43,925,452 (92.96%)
Protrudent 1	43,242,926	43,136,608 (99.75%)	98.06%	94.18%	46.16%	40,063,778 (92.94%)
Protrudent 2	44,343,242	44,233,162 (99.75%)	97.85%	93.68%	46.24%	41,018,154 (92.79%)
Protrudent 3	48,156,128	48,026,282 (99.73%)	97.88%	93.78%	46.18%	44,482,464 (92.71%)

## Data Availability

Not applicable.

## Supplementary Materials

The following supporting information can be downloaded at: https://www.mdpi.com/article/10.3390/genes14091785/s1, Figure S1: The phenotype of protrudent fruit dots bulged and exhibited a white ring around the fruit dots; Table S1: The information of several structural genes involved in lignin biosynthesis in pear; Table S2: The information of several regulatory genes involved in lignin biosynthesis in pear; Table S3: The primers used for RT-qPCR analysis in the experiment.
